# Ethyl 2-[*N*-(2-Formyl­phen­yl)benzene­sulfonamido]acetate

**DOI:** 10.1107/S1600536809004413

**Published:** 2009-02-18

**Authors:** P. R. Seshadri, B. Balakrishnan, K. Ilangovan, R. Sureshbabu, A. K. Mohanakrishnan

**Affiliations:** a PG and Research Department of Physics, Agurchand Manmull Jain College, Chennai 600 114, India; b Department of Physics, P. T. Lee Chengalvaraya Naicker College of Engineering and Technology, Kancheepuram 631 502, India; c PG and Research Department of Physics, RKM Vivekananda College, Chennai 600 004, India; d Department of Organic Chemistry, University of Madras, Guindy Campus, Chennai 600 025, India

## Abstract

In the title compound, C_17_H_17_NO_5_S, the N atom is *sp* ^3^-hybridized and the S atom has a distorted tetra­hedral configuration. The dihedral angle between the two aromatic rings is 30.0 (1)°, and that between the ethyl acetate group and the formyl­phenyl ring is 77.4 (1)°. The mol­ecules are linked into chains along [100] by C—H⋯O hydrogen bonds and the chains are linked *via* C—H⋯π inter­actions.

## Related literature

For the biological properties of sulfonamide derivatives, see: Brown (1971 ▶); Nieto *et al.* (2005 ▶); Pomarnacka & Kozlarska-Kedra (2003 ▶). For related structures, see: Cameron *et al.* (1975 ▶); Cotton & Stokley (1970 ▶); Usha *et al.* (2005 ▶); Zhu *et al.* (2008 ▶).
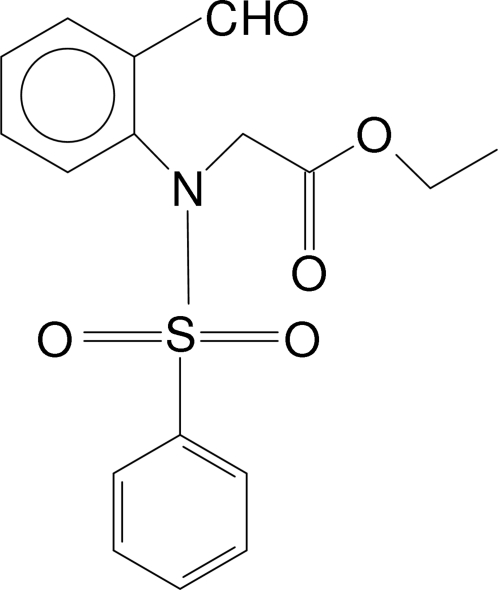


## Experimental

### 

#### Crystal data

C_17_H_17_NO_5_S
*M* *_r_* = 347.38
Orthorhombic, 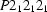

*a* = 11.3442 (5) Å
*b* = 11.7731 (6) Å
*c* = 12.7809 (6) Å
*V* = 1706.97 (14) Å^3^
*Z* = 4
Mo *K*α radiation
μ = 0.22 mm^−1^
*T* = 293 K
0.25 × 0.20 × 0.20 mm

#### Data collection

Bruker Kappa APEXII area-detector diffractometer
Absorption correction: multi-scan (*SADABS*; Sheldrick, 2001 ▶) *T*_min_ = 0.948, *T*_max_ = 0.958
10886 measured reflections
4104 independent reflections
3294 reflections with *I* > 2σ(*I*)
*R*_int_ = 0.022

#### Refinement

*R*[*F* ^2^ > 2σ(*F* ^2^)] = 0.036
*wR*(*F* ^2^) = 0.092
*S* = 0.95
4104 reflections
218 parameters
H-atom parameters constrained
Δρ_max_ = 0.31 e Å^−3^
Δρ_min_ = −0.23 e Å^−3^
Absolute structure: Flack (1983 ▶), 1714 Friedel pairs
Flack parameter: −0.05 (7)

### 

Data collection: *APEX2* (Bruker, 2004 ▶); cell refinement: *SAINT* (Bruker, 2004 ▶); data reduction: *SAINT*; program(s) used to solve structure: *SHELXS97* (Sheldrick, 2008 ▶); program(s) used to refine structure: *SHELXL97* (Sheldrick, 2008 ▶); molecular graphics: *ORTEP-3* (Farrugia, 1997 ▶) and *PLATON* (Spek, 2009 ▶); software used to prepare material for publication: *SHELXL97*.

## Supplementary Material

Crystal structure: contains datablocks I, global. DOI: 10.1107/S1600536809004413/ci2763sup1.cif

Structure factors: contains datablocks I. DOI: 10.1107/S1600536809004413/ci2763Isup2.hkl

Additional supplementary materials:  crystallographic information; 3D view; checkCIF report

## Figures and Tables

**Table 1:** Hydrogen-bond geometry (Å, °)

*D*—H⋯*A*	*D*—H	H⋯*A*	*D*⋯*A*	*D*—H⋯*A*
C8—H8⋯O3^i^	0.93	2.57	3.218 (3)	127
C16—H16*B*⋯*Cg*1^ii^	0.97	2.73	3.605 (3)	150

Symmetry codes: (i) 
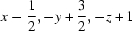
; (ii) 
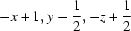
. *Cg*1 is the centroid of C1–C6 ring.

**Fig. 1.**
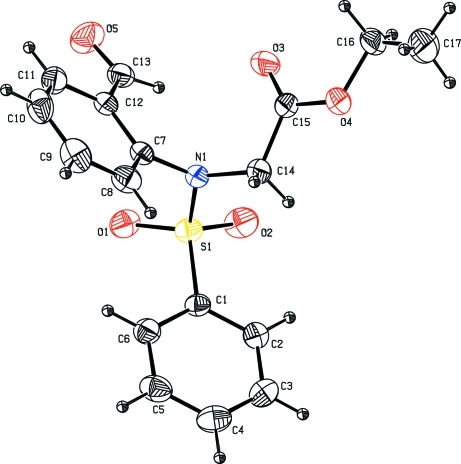
Molecular structure of the title compound, showing 30% probability displacement ellipsoids.

**Fig. 2.**
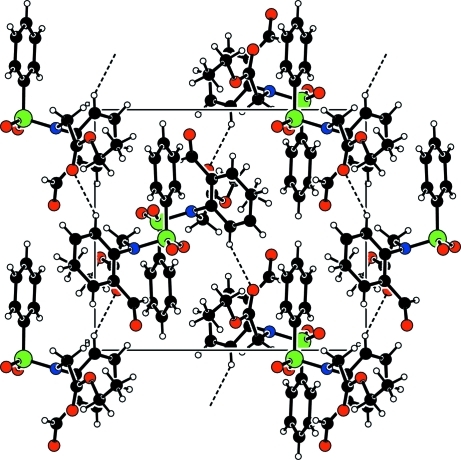
Crystal packing of the title compound. Hydrogen bonds are shown as dashed lines.

## supplementary crystallographic information

### Comment

Sulfonamide derivates are well known drugs and are used to control diseases caused by bacterial infections. The antibacterial action of this group of drugs is exerted by the complete inhibition of dihydropteroate synthase enzyme towards the *p*-amino benzonate (Brown, 1971). Benzene sulfonamide derivatives are known to exhibit anticancer and HIV activities (Pomarnacka & Kozlarska-Kedra, 2003) and antibacterial activities (Nieto *et al.*, 2005). In view of this medicinal importance, the crystal structure determination of the title compound (Fig.1) was carried out and the results are presented here.

The angles around atom S1 deviate significantly from the regular tetrahedral value, with the largest deviation of 120.6 (1)° for O1—S1—O2 angle. This may be due to non-bonding interactions between S═O bonds (Cotton & Stokley, 1970). The sulfonyl oxygen O1 is syn-clinal and O2 is syn-periplanar to the phenyl ring. The dihedral angle between the best planes through the ethylacetate group (O3/O4/C14/C15/C16) and formyl phenyl ring (C7-C12) is 77.4 (1)°. The aldyhyde group is slightly twisted from the plane of the ring to which it is attached as evidenced by the torsion angle C11—C12—C13—O5 of -8.5 (3)°. The relative orientations of C/N/S and O/S/O planes is determined by the hybridization nature of atom N1. The angles between planes C14/N1/S1 and O1/S1/O2 and between C7/N1/S1 and O1/S1/O2 planes are 59° and 56°, respectively. These values are close to 58° reported for *sp*^3^ N atoms [87° for *sp*^2^ N atoms] (Cameron *et al.*, 1975). The geometrical parameters agree well with those reported for related sulfonamide structures (Usha *et al.*, 2005; Zhu *et al.*, 2008).

In addition to van der Waals interactions, the crystal structure is stabilized by C—H···O and C—H···π interactions (Table 1).

### Experimental

N-(2-Formylphenyl)benzene sulfonamide (1.7 g, 6.5 mmol) was dissolved in dimethyl acetamide (25 ml). To this potassium carbonate (2.25 g, 16.2 mmol) and ethyl bromoacetate (0.86 ml, 7.8 mmol) were added. The reaction mixture was stirred at room temperature for 4 h. It was then poured over curshed ice (100 g) cotaining 5 ml of concentrated HCl. The precipitated solid was filtered off and the title compound was recrystallized from methanol. Yield = 1 g (45%) and m.p = 381 K.

### Refinement

All H atoms were positioned geometrically and allowed to ride on their parent atoms, with C-H = 0.93-0.97 Å and U_iso_(H) = 1.5*U*_eq_(C) for methyl H atoms and 1.2*U*_eq_(C) for other H atoms.

### Crystal data

**Table:**

C_17_H_17_NO_5_S	*F*(000) = 728
*M**_r_* = 347.38	*D*_x_ = 1.352 Mg m^−^^3^
Orthorhombic, *P*2_1_2_1_2_1_	Mo *K*α radiation, λ = 0.71073 Å
Hall symbol: P 2ac 2ab	Cell parameters from 4197 reflections
*a* = 11.3442 (5) Å	θ = 2.4–28.2°
*b* = 11.7731 (6) Å	µ = 0.22 mm^−^^1^
*c* = 12.7809 (6) Å	*T* = 293 K
*V* = 1706.97 (14) Å^3^	Block, colourless
*Z* = 4	0.25 × 0.20 × 0.20 mm

### Data collection

**Table:**

Bruker Kappa APEXII area-detector diffractometer	4104 independent reflections
Radiation source: fine-focus sealed tube	3294 reflections with *I* > 2σ(*I*)
graphite	*R*_int_ = 0.022
ω scans	θ_max_ = 28.2°, θ_min_ = 2.4°
Absorption correction: multi-scan (*SADABS*; Sheldrick, 2001)	*h* = −15→10
*T*_min_ = 0.948, *T*_max_ = 0.958	*k* = −13→15
10886 measured reflections	*l* = −17→17

### Refinement

**Table:**

Refinement on *F*^2^	Secondary atom site location: difference Fourier map
Least-squares matrix: full	Hydrogen site location: inferred from neighbouring sites
*R*[*F*^2^ > 2σ(*F*^2^)] = 0.036	H-atom parameters constrained
*wR*(*F*^2^) = 0.092	*w* = 1/[σ^2^(*F*_o_^2^) + (0.0478*P*)^2^ + 0.2327*P*] where *P* = (*F*_o_^2^ + 2*F*_c_^2^)/3
*S* = 0.95	(Δ/σ)_max_ = 0.001
4104 reflections	Δρ_max_ = 0.31 e Å^−^^3^
218 parameters	Δρ_min_ = −0.23 e Å^−^^3^
0 restraints	Absolute structure: Flack (1983), 1714 Friedel pairs
Primary atom site location: structure-invariant direct methods	Flack parameter: −0.05 (7)

### Special details

**Table:**

Geometry. All e.s.d.'s (except the e.s.d. in the dihedral angle between two l.s. planes) are estimated using the full covariance matrix. The cell e.s.d.'s are taken into account individually in the estimation of e.s.d.'s in distances, angles and torsion angles; correlations between e.s.d.'s in cell parameters are only used when they are defined by crystal symmetry. An approximate (isotropic) treatment of cell e.s.d.'s is used for estimating e.s.d.'s involving l.s. planes.
Refinement. Refinement of *F*^2^ against ALL reflections. The weighted *R*-factor *wR* and goodness of fit *S* are based on *F*^2^, conventional *R*-factors *R* are based on *F*, with *F* set to zero for negative *F*^2^. The threshold expression of *F*^2^ > σ(*F*^2^) is used only for calculating *R*-factors(gt) *etc*. and is not relevant to the choice of reflections for refinement. *R*-factors based on *F*^2^ are statistically about twice as large as those based on *F*, and *R*- factors based on ALL data will be even larger.

### Fractional atomic coordinates and isotropic or equivalent isotropic displacement parameters (Å^2^)

**Table:**

	*x*	*y*	*z*	*U*_iso_*/*U*_eq_	
S1	0.53613 (4)	0.77486 (4)	0.23609 (3)	0.04875 (13)	
N1	0.57693 (13)	0.74419 (13)	0.35602 (12)	0.0458 (4)	
O1	0.58109 (13)	0.88549 (13)	0.21568 (11)	0.0627 (4)	
O2	0.56980 (14)	0.68125 (16)	0.17264 (13)	0.0734 (5)	
O3	0.75001 (14)	0.59352 (14)	0.41673 (16)	0.0749 (5)	
O4	0.61531 (12)	0.47431 (12)	0.48118 (11)	0.0576 (4)	
O5	0.89664 (14)	0.90059 (16)	0.34906 (17)	0.0833 (6)	
C1	0.38089 (15)	0.77875 (16)	0.23788 (14)	0.0433 (4)	
C2	0.3190 (2)	0.68510 (18)	0.20483 (17)	0.0559 (5)	
H2	0.3585	0.6215	0.1798	0.067*	
C3	0.1975 (2)	0.6866 (2)	0.2093 (2)	0.0693 (6)	
H3	0.1546	0.6237	0.1870	0.083*	
C4	0.1400 (2)	0.7802 (2)	0.24649 (19)	0.0705 (6)	
H4	0.0581	0.7806	0.2496	0.085*	
C5	0.2021 (2)	0.8738 (2)	0.27923 (18)	0.0660 (6)	
H5A	0.1622	0.9371	0.3046	0.079*	
C6	0.32367 (19)	0.87418 (18)	0.27450 (16)	0.0547 (5)	
H6	0.3663	0.9376	0.2956	0.066*	
C7	0.60070 (16)	0.83376 (15)	0.42938 (14)	0.0430 (4)	
C8	0.51433 (19)	0.86705 (19)	0.49933 (16)	0.0585 (5)	
H8	0.4400	0.8338	0.4967	0.070*	
C9	0.5382 (3)	0.9487 (2)	0.57219 (17)	0.0717 (6)	
H9	0.4799	0.9708	0.6192	0.086*	
C10	0.6472 (3)	0.9983 (2)	0.57653 (17)	0.0713 (7)	
H10	0.6630	1.0529	0.6273	0.086*	
C11	0.7342 (2)	0.96779 (17)	0.50592 (16)	0.0585 (5)	
H11	0.8075	1.0030	0.5082	0.070*	
C12	0.71158 (17)	0.88403 (15)	0.43139 (14)	0.0444 (4)	
C13	0.80492 (18)	0.85128 (18)	0.35747 (16)	0.0525 (5)	
H13	0.7919	0.7886	0.3148	0.063*	
C14	0.54423 (19)	0.63341 (16)	0.39786 (16)	0.0529 (4)	
H14A	0.5022	0.5908	0.3447	0.064*	
H14B	0.4917	0.6437	0.4570	0.064*	
C15	0.65033 (19)	0.56723 (17)	0.43213 (15)	0.0485 (4)	
C16	0.7055 (2)	0.39708 (18)	0.51686 (18)	0.0642 (6)	
H16A	0.7631	0.4372	0.5591	0.077*	
H16B	0.7457	0.3630	0.4577	0.077*	
C17	0.6461 (3)	0.3085 (2)	0.5799 (3)	0.1089 (12)	
H17A	0.6111	0.3426	0.6406	0.163*	
H17B	0.7027	0.2525	0.6012	0.163*	
H17C	0.5858	0.2729	0.5386	0.163*	

### Atomic displacement parameters (Å^2^)

**Table:**

	*U*^11^	*U*^22^	*U*^33^	*U*^12^	*U*^13^	*U*^23^
S1	0.0432 (2)	0.0579 (3)	0.0451 (2)	0.0008 (2)	0.0015 (2)	−0.0029 (2)
N1	0.0426 (8)	0.0414 (8)	0.0535 (8)	0.0000 (7)	−0.0064 (6)	0.0046 (7)
O1	0.0607 (9)	0.0714 (10)	0.0559 (8)	−0.0149 (8)	−0.0027 (7)	0.0166 (7)
O2	0.0594 (10)	0.0918 (12)	0.0692 (9)	0.0096 (9)	0.0088 (7)	−0.0290 (9)
O3	0.0449 (9)	0.0622 (10)	0.1175 (14)	0.0016 (8)	−0.0120 (9)	0.0194 (9)
O4	0.0636 (9)	0.0468 (8)	0.0622 (8)	0.0010 (7)	−0.0076 (7)	0.0095 (7)
O5	0.0478 (9)	0.0929 (13)	0.1093 (14)	−0.0136 (9)	0.0124 (9)	−0.0077 (11)
C1	0.0411 (9)	0.0509 (10)	0.0380 (8)	0.0016 (8)	−0.0043 (7)	−0.0017 (9)
C2	0.0555 (13)	0.0491 (11)	0.0630 (12)	0.0033 (10)	−0.0050 (9)	−0.0037 (9)
C3	0.0548 (13)	0.0680 (14)	0.0852 (16)	−0.0116 (12)	−0.0057 (12)	−0.0013 (12)
C4	0.0466 (11)	0.0937 (18)	0.0712 (14)	0.0004 (13)	0.0025 (10)	0.0003 (14)
C5	0.0592 (13)	0.0776 (15)	0.0612 (13)	0.0218 (13)	−0.0023 (11)	−0.0129 (12)
C6	0.0576 (12)	0.0564 (12)	0.0502 (10)	0.0069 (10)	−0.0082 (9)	−0.0095 (9)
C7	0.0454 (10)	0.0408 (9)	0.0429 (9)	0.0049 (8)	−0.0035 (8)	0.0058 (8)
C8	0.0522 (12)	0.0656 (13)	0.0576 (11)	0.0071 (10)	0.0076 (9)	0.0062 (10)
C9	0.0859 (17)	0.0712 (14)	0.0579 (12)	0.0135 (15)	0.0170 (14)	−0.0028 (11)
C10	0.111 (2)	0.0521 (12)	0.0508 (12)	0.0063 (13)	−0.0067 (13)	−0.0087 (10)
C11	0.0719 (15)	0.0488 (11)	0.0547 (12)	−0.0025 (11)	−0.0150 (10)	0.0027 (9)
C12	0.0463 (10)	0.0420 (9)	0.0449 (9)	0.0045 (8)	−0.0050 (8)	0.0067 (8)
C13	0.0432 (11)	0.0538 (11)	0.0605 (12)	0.0035 (10)	−0.0014 (9)	0.0079 (9)
C14	0.0452 (11)	0.0452 (10)	0.0684 (11)	−0.0022 (10)	−0.0031 (10)	0.0073 (9)
C15	0.0523 (12)	0.0427 (10)	0.0505 (10)	−0.0004 (9)	−0.0091 (9)	−0.0037 (8)
C16	0.0804 (16)	0.0486 (11)	0.0635 (13)	0.0096 (11)	−0.0243 (11)	0.0018 (10)
C17	0.132 (3)	0.0725 (18)	0.122 (3)	−0.0193 (18)	−0.047 (2)	0.0430 (18)

### Geometric parameters (Å, °)

**Table:**

S1—O1	1.4229 (15)	C7—C8	1.383 (3)
S1—O2	1.4206 (16)	C7—C12	1.390 (3)
S1—N1	1.6414 (15)	C8—C9	1.365 (3)
S1—C1	1.7618 (18)	C8—H8	0.93
N1—C7	1.437 (2)	C9—C10	1.370 (4)
N1—C14	1.458 (2)	C9—H9	0.93
O3—C15	1.189 (2)	C10—C11	1.385 (3)
O4—C15	1.322 (2)	C10—H10	0.93
O4—C16	1.443 (2)	C11—C12	1.395 (3)
O5—C13	1.196 (3)	C11—H11	0.93
C1—C6	1.379 (3)	C12—C13	1.471 (3)
C1—C2	1.374 (3)	C13—H13	0.93
C2—C3	1.380 (3)	C14—C15	1.499 (3)
C2—H2	0.93	C14—H14A	0.97
C3—C4	1.367 (3)	C14—H14B	0.97
C3—H3	0.93	C16—C17	1.480 (4)
C4—C5	1.373 (3)	C16—H16A	0.97
C4—H4	0.93	C16—H16B	0.97
C5—C6	1.381 (3)	C17—H17A	0.96
C5—H5A	0.93	C17—H17B	0.96
C6—H6	0.93	C17—H17C	0.96
			
O1—S1—O2	120.6 (1)	C8—C9—H9	119.8
O1—S1—N1	105.7 (1)	C10—C9—H9	119.8
O2—S1—N1	106.7 (1)	C9—C10—C11	120.4 (2)
O1—S1—C1	109.7 (1)	C9—C10—H10	119.8
O2—S1—C1	107.2 (1)	C11—C10—H10	119.8
N1—S1—C1	106.0 (1)	C10—C11—C12	119.8 (2)
C7—N1—C14	117.7 (2)	C10—C11—H11	120.1
C7—N1—S1	120.1 (1)	C12—C11—H11	120.1
C14—N1—S1	117.9 (1)	C7—C12—C11	118.69 (19)
C15—O4—C16	117.27 (16)	C7—C12—C13	121.86 (18)
C6—C1—C2	121.17 (18)	C11—C12—C13	119.45 (19)
C6—C1—S1	119.73 (15)	O5—C13—C12	123.8 (2)
C2—C1—S1	119.08 (15)	O5—C13—H13	118.1
C3—C2—C1	119.2 (2)	C12—C13—H13	118.1
C3—C2—H2	120.4	N1—C14—C15	111.6 (2)
C1—C2—H2	120.4	N1—C14—H14A	109.3
C2—C3—C4	120.1 (2)	C15—C14—H14A	109.3
C2—C3—H3	120.0	N1—C14—H14B	109.3
C4—C3—H3	120.0	C15—C14—H14B	109.3
C5—C4—C3	120.6 (2)	H14A—C14—H14B	108.0
C5—C4—H4	119.7	O3—C15—O4	125.4 (2)
C3—C4—H4	119.7	O3—C15—C14	125.5 (2)
C4—C5—C6	120.1 (2)	O4—C15—C14	109.11 (17)
C4—C5—H5A	120.0	O4—C16—C17	107.0 (2)
C6—C5—H5A	120.0	O4—C16—H16A	110.3
C1—C6—C5	118.9 (2)	C17—C16—H16A	110.3
C1—C6—H6	120.6	O4—C16—H16B	110.3
C5—C6—H6	120.6	C17—C16—H16B	110.3
C8—C7—C12	120.55 (19)	H16A—C16—H16B	108.6
C8—C7—N1	119.79 (18)	C16—C17—H17A	109.5
C12—C7—N1	119.63 (17)	C16—C17—H17B	109.5
C9—C8—C7	120.0 (2)	H17A—C17—H17B	109.5
C9—C8—H8	120.0	C16—C17—H17C	109.5
C7—C8—H8	120.0	H17A—C17—H17C	109.5
C8—C9—C10	120.5 (2)	H17B—C17—H17C	109.5
			
O1—S1—N1—C7	24.90 (17)	C14—N1—C7—C12	119.11 (18)
O2—S1—N1—C7	154.43 (15)	S1—N1—C7—C12	−84.75 (19)
C1—S1—N1—C7	−91.53 (15)	C12—C7—C8—C9	−1.0 (3)
O1—S1—N1—C14	−179.00 (14)	N1—C7—C8—C9	177.16 (19)
O2—S1—N1—C14	−49.47 (17)	C7—C8—C9—C10	0.1 (3)
C1—S1—N1—C14	64.58 (16)	C8—C9—C10—C11	1.1 (4)
O1—S1—C1—C6	−33.51 (18)	C9—C10—C11—C12	−1.5 (3)
O2—S1—C1—C6	−166.12 (17)	C8—C7—C12—C11	0.6 (3)
N1—S1—C1—C6	80.23 (17)	N1—C7—C12—C11	−177.55 (16)
O1—S1—C1—C2	148.00 (16)	C8—C7—C12—C13	−179.43 (17)
O2—S1—C1—C2	15.4 (2)	N1—C7—C12—C13	2.4 (3)
N1—S1—C1—C2	−98.26 (16)	C10—C11—C12—C7	0.6 (3)
C6—C1—C2—C3	−0.5 (3)	C10—C11—C12—C13	−179.32 (19)
S1—C1—C2—C3	177.94 (18)	C7—C12—C13—O5	171.5 (2)
C1—C2—C3—C4	−0.2 (3)	C11—C12—C13—O5	−8.5 (3)
C2—C3—C4—C5	0.3 (4)	C7—N1—C14—C15	−80.8 (2)
C3—C4—C5—C6	0.2 (4)	S1—N1—C14—C15	122.49 (16)
C2—C1—C6—C5	1.1 (3)	C16—O4—C15—O3	−1.5 (3)
S1—C1—C6—C5	−177.41 (17)	C16—O4—C15—C14	177.78 (17)
C4—C5—C6—C1	−0.9 (3)	N1—C14—C15—O3	−8.5 (3)
C14—N1—C7—C8	−59.1 (2)	N1—C14—C15—O4	172.19 (15)
S1—N1—C7—C8	97.09 (19)	C15—O4—C16—C17	173.1 (2)

### Hydrogen-bond geometry (Å, °)

**Table:**

*D*—H···*A*	*D*—H	H···*A*	*D*···*A*	*D*—H···*A*
C8—H8···O3^i^	0.93	2.57	3.218 (3)	127
C16—H16B···Cg1^ii^	0.97	2.73	3.605 (3)	150

Symmetry codes: (i) *x*−1/2, −*y*+3/2, −*z*+1; (ii) −*x*+1, *y*−1/2, −*z*+1/2.
